# The impact of updated imaging software on the performance of machine learning models for breast cancer diagnosis: a multi-center, retrospective study

**DOI:** 10.1007/s00404-024-07901-8

**Published:** 2025-01-30

**Authors:** Lie Cai, Michael Golatta, Chris Sidey-Gibbons, Richard G. Barr, André Pfob

**Affiliations:** 1 Department of Obstetrics and Gynecology, Breast Cancer Center, Heidelberg University Hospital, Im Neuenheimer Feld 440, 69120 Heidelberg, Germany; 2 Breast Centre Heidelberg, Klinik St. Elisabeth, Heidelberg, Germany; 3 MD Anderson Center for INSPiRED Cancer Care (Integrated Systems for Patient-Reported Data), The University of Texas MD Anderson Cancer Center, Houston, TX USA; 4 Department of Symptom Research, The University of Texas MD Anderson Cancer Center, Houston, TX USA; 5 Department of Radiology, Northeast Ohio Medical University, Ravenna, OH USA; 6 National Center for Tumor Diseases (NCT) and German Cancer Research Center (DKFZ), Heidelberg, Germany

**Keywords:** Machine learning, Medical imaging, Breast, Shear wave elastography

## Abstract

**Purpose:**

Artificial Intelligence models based on medical (imaging) data are increasingly developed. However, the imaging software on which the original data is generated is frequently updated. The impact of updated imaging software on the performance of AI models is unclear. We aimed to develop machine learning models using shear wave elastography (SWE) data to identify malignant breast lesions and to test the models’ generalizability by validating them on external data generated by both the original updated software versions.

**Methods:**

We developed and validated different machine learning models (GLM, MARS, XGBoost, SVM) using multicenter, international SWE data (NCT 02638935) using tenfold cross-validation. Findings were compared to the histopathologic evaluation of the biopsy specimen or 2-year follow-up. The outcome measure was the area under the curve (AUROC).

**Results:**

We included 1288 cases in the development set using the original imaging software and 385 cases in the validation set using both, original and updated software. In the external validation set, the GLM and XGBoost models showed better performance with the updated software data compared to the original software data (AUROC 0.941 vs. 0.902, *p* < 0.001 and 0.934 vs. 0.872, *p* < 0.001). The MARS model showed worse performance with the updated software data (0.847 vs. 0.894, *p* = 0.045). SVM was not calibrated.

**Conclusion:**

In this multicenter study using SWE data, some machine learning models demonstrated great potential to bridge the gap between original software and updated software, whereas others exhibited weak generalizability.

**Supplementary Information:**

The online version contains supplementary material available at 10.1007/s00404-024-07901-8.

## What does this study add to the clinical work

**Table:** 

When developing machine learning models using medical imaging data, the impact of data drift should be systematically evaluated to maintain model reliability over time. Some machine learning models demonstrated great potential to bridge the gap between original and updated software, whereas others exhibited weak generalizability.	

## Introduction

The U.S. Food and Drug Administration (FDA) has approved 692 artificial intelligence (AI) and machine learning (ML) algorithms (as of October 2023) [1]. AI has demonstrated great potential in medical diagnosis and treatment decision-making [2, 3]. However, the performance of robust AI models frequently decline, and in some cases, they fail during long-term prospective validation [4]. Beyond the classical limitations associated with these models, such as biases in data selection [5] and the lack of diversity in ethical and demographic representation [6], there exists a critical and often overlooked challenge that warrants further attention from researchers: while new AI imaging models are rapidly developed, the imaging software that generates the data is being updated frequently which may lead to data drift. However, the impact of updated imaging software on the performance of AI algorithms as well as the methods for monitoring such potential data drift is unclear [7].

Shear wave elastography (SWE) is an ultrasound technique measuring the stiffness of a mass to improve the diagnostic performance of B-mode ultrasound [8]. Recently, the FDA approved a new software for breast ultrasound SWE [9]. The new software generates substantially different data compared to the original software version, as it expands the range of shear wave velocity, which improves benign versus malignant lesion differentiation and the display of very stiff and very small breast lesions [9]. A previous study has shown that an intelligent SWE model based on imaging data generated by the old software can reliably classify breast lesions and reduce unnecessary biopsies [10]. Whether the performance of such models remains stable when using the new software version, is unknown.

We aimed to evaluate the impact of updated medical imaging software on data drift and the generalizability of AI models by developing machine learning models using the original SWE data to identify malignant breast lesions and validating them on external data generated by both the original and updated software versions.

## Materials and methods

### Study design

This multi-center, retrospective study was conducted in accordance with the Declaration of Helsinki and was approved by the Ethics Committee of Heidelberg University Medical Faculty.

In this study, we aimed to develop machine learning models using SWE data to identify malignant breast lesions and to test the models’ generalizability by validating them on external data generated by the original software version and an updated new software version. We used multi-center international SWE data (NCT 02638935) as the development set. Women aged 18 years or older who presented with a suspicious or indeterminate single breast mass of diameters between 0.5 and 5 cm in B-mode ultrasound from February 2016 to March 2019 were included in this study [10]. We used SWE data from a single-center study as the validation set. This observational study included patients scheduled for a screening or diagnostic breast ultrasound from April 25, 2019, to May 2, 2022 [9]. In the development set, shear wave velocity (SWV, in m/s) was measured three times consecutively by a board-certified physician using the original software at the area of highest SWV within the mass. In the validation set, SWV was measured two times using the original software and updated software separately by a board-certified physician. The third SWV measurement was generated by calculating the mean value of the previous two measurements.

### Outcome and definitions

Pathological evaluation of the breast biopsy specimen or > 2-year follow-up served as the gold standard for the dignity of the breast masses. All malignant lesions were biopsy-proven.

### Model construction and evaluation

For the algorithm development and reporting, we considered guidelines and best-practice papers on machine learning in medicine [11, 12], diagnostic tests [13], and multivariable prediction models [14]. A checklist informed by recent guidelines on machine learning in medicine is provided in the Data Supplement.

**Fig. 1:**
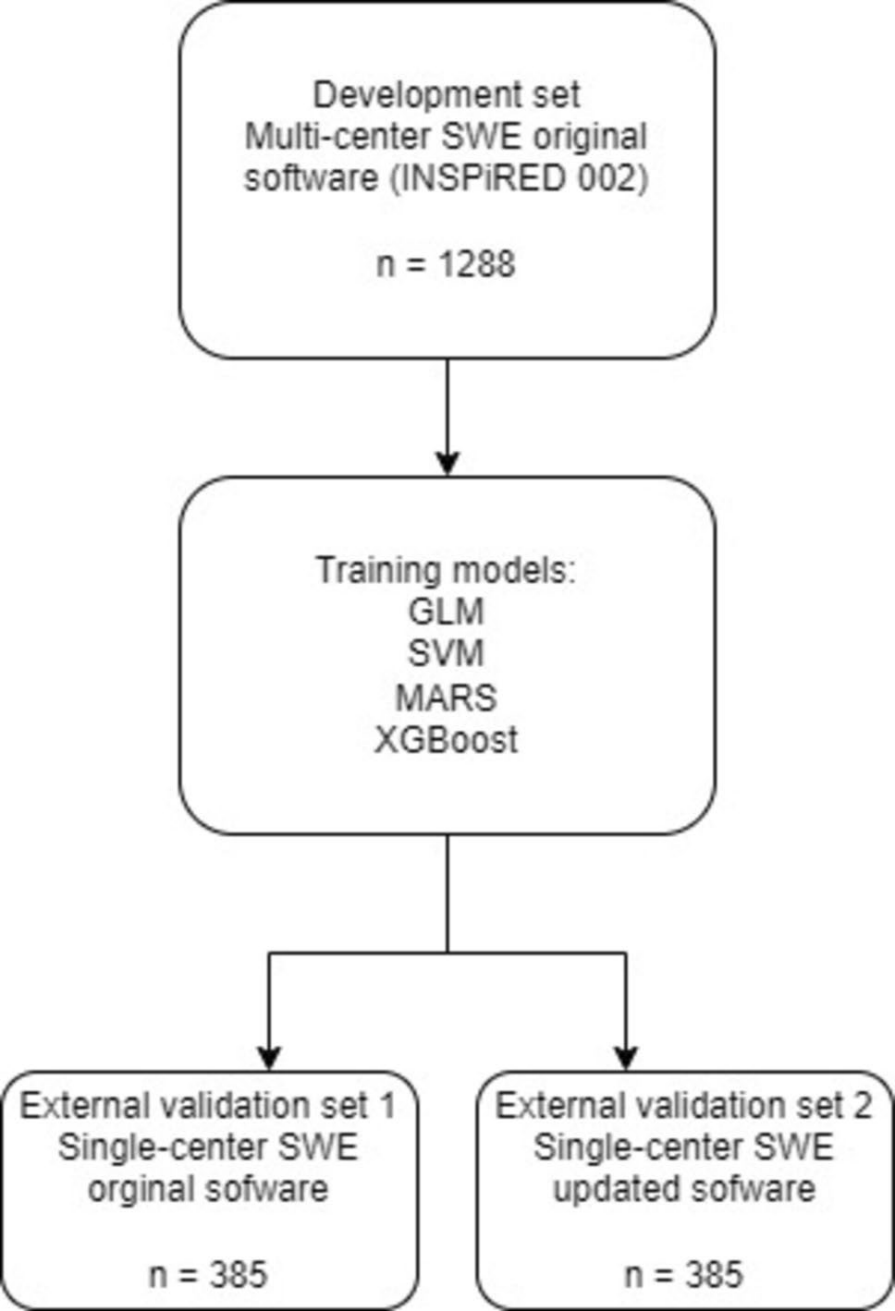
Diagram of patient flow

We chose logistic regression with the elastic net penalty (GLM), motivation, ability, role perceptions, and situational factors (MARS), support vector machine (SVM), and extreme gradient boosting (XGBoost) algorithms for model construction. tenfold cross-validation was used for the algorithm training and internal testing on the development dataset. A hypergrid search was performed to select the optimal hyperparameters for each algorithm. The area under the receiver operating characteristic curve (AUC) was considered the main measurement of model performance. Final models were then validated using the external validation set (Fig. 1).

We used the “DALEX” package in R to calculate the agnostic variable importance measure computed via permutation (e.g. computing the loss function for the full model and then computing randomized response variables’ loss function). We used decision curve analysis (DCA) to illustrate the benefits of the clinical application of the models [15]. Python (Version 3.11.5) and R (Version 4.3.1) were used for all analyses.

### Statistical analysis

We performed a descriptive analysis to illustrate the distribution of the baseline characteristics of the development set and the external validation set. We used the Chi-square test for categorical data, and the *t *test for continuous data to compare differences in baseline characteristics between the development and validation set. We calculated AUC values and accompanying 95% CIs for the algorithms using 2000 bootstrap replicates stratified for the outcome variable (malignant and benign). The Venkatraman method tests were used to compare models’ performance [16]. Proportion test was used to compare the model’s diagnostic performance [17]. Calibration plots (observed *vs.* predicted probabilities) and Spiegelhalter’s Z statistics were used to evaluate model calibration [18, 19].

We considered *p* values < 0.05 to be statistically significant.

## Results

### Patient flow

**Fig. 2:**
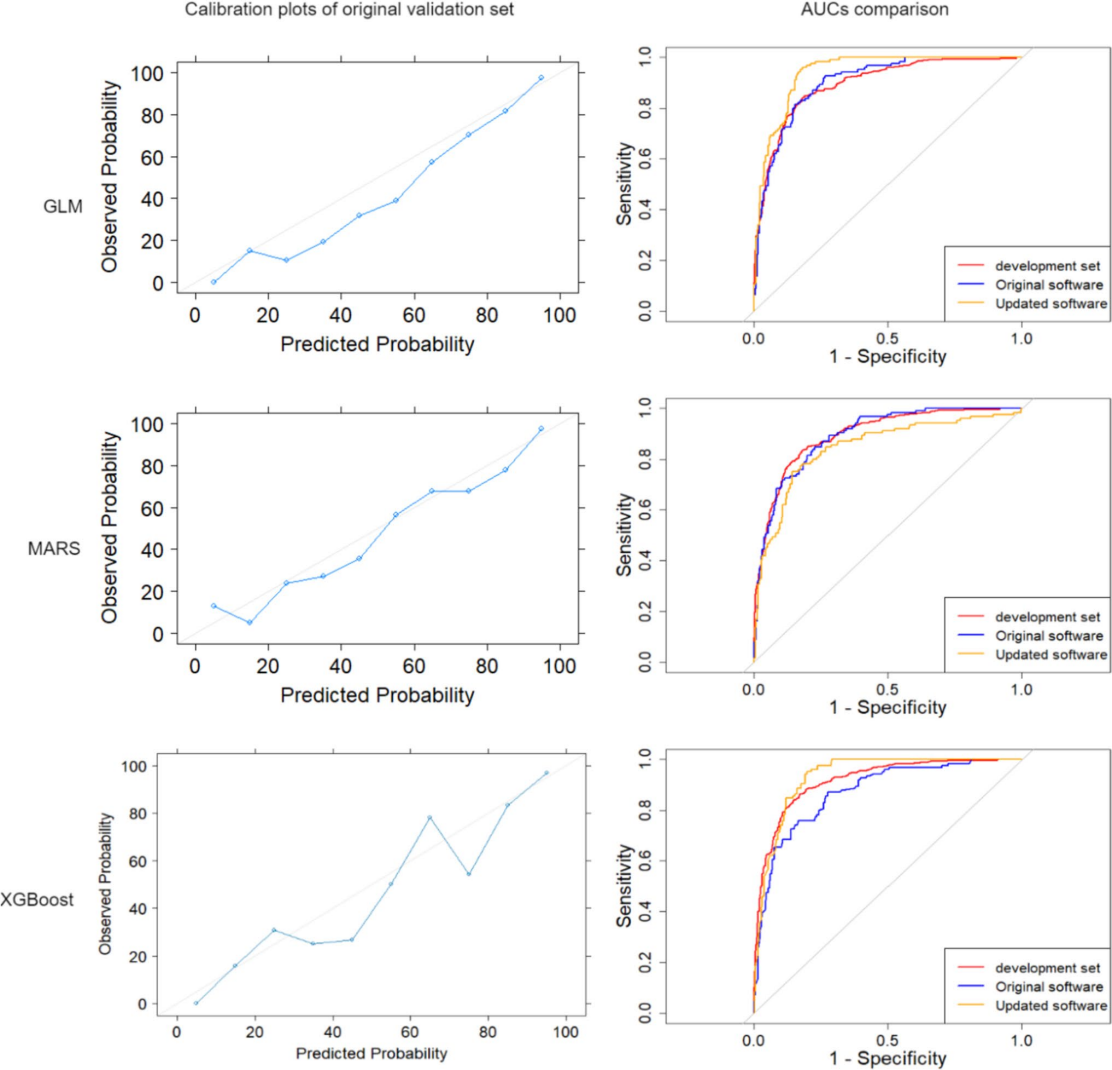
Models’ calibration plots of original software in the external validation set and AUC curves. *AUC* area under the curve, *GLM* logistic regression with elastic net penalty, *MARS* motivation, ability, role perceptions, situational factors, *XGBoost* extreme gradient boosting

We used an international, multicenter SWE study (NCT 02638935) [10] as the development set, which included 1288 patients and where data were obtained using the original imaging software. We used another single-center SWE study [9] as the external validation set, which included 385 patients and where data was obtained using both, the original software and the new updated software (Figs. 1, 2).

### Baseline characteristics

Of the 1288 patients in the development set, 28.6% (368 of 1288) had a malignant breast mass. In the external validation set. 32.2% (124 of 385) had a malignant breast mass. Patients in the external validation set were older (mean age, 54.81 *vs.* 46.49, *p* < 0.001) and had a larger lesion size (15.65 mm vs. 11.90 mm, *p* < 0.001) compared to the development set. The shear wave velocity measured in the external validation set was significantly different compared to the development set for both, the original software and the updated software (*p* < 0.001). Details regarding baseline clinical characteristics are shown in Table 1.

**Table 1:** Baseline clinical characteristics comparison between the development set and external validation set

Characteristics	Development set (*n* = 1288)	Validation set (*n* = 385)			*p*
Age (SD)	46.49 (16.05)	54.81 (16.20)			< 0.001*
Lesion, no. (%)					0.169
Malignant	368 (28.6)	124 (32.2)			
Benign	920 (71.4)	261 (67.8)			
Lesion size (SD)	15.65 (7.31)	11.90 (8.03)			< 0.001*
		Original software	*p*	Updated software	*p*
Shear Wave Velocity, measurement 1–m/s (SD)	4.02 (2.08)	2.95 (1.80)	< 0.001*	4.80 (3.02)	< 0.001*
Shear Wave Velocity, measurement 2–m/s (SD)	4.04 (2.08)	3.12 (1.80)		5.06 (3.06)	
Shear Wave Velocity, measurement 3–m/s (SD)	4.06 (2.15)	3.03 (1.80)		4.93 (3.04)	

*SD* standard deviation, *SWV* shear wave velocity

*Statistical significance

### Model calibration and performance

**Fig. 3:**
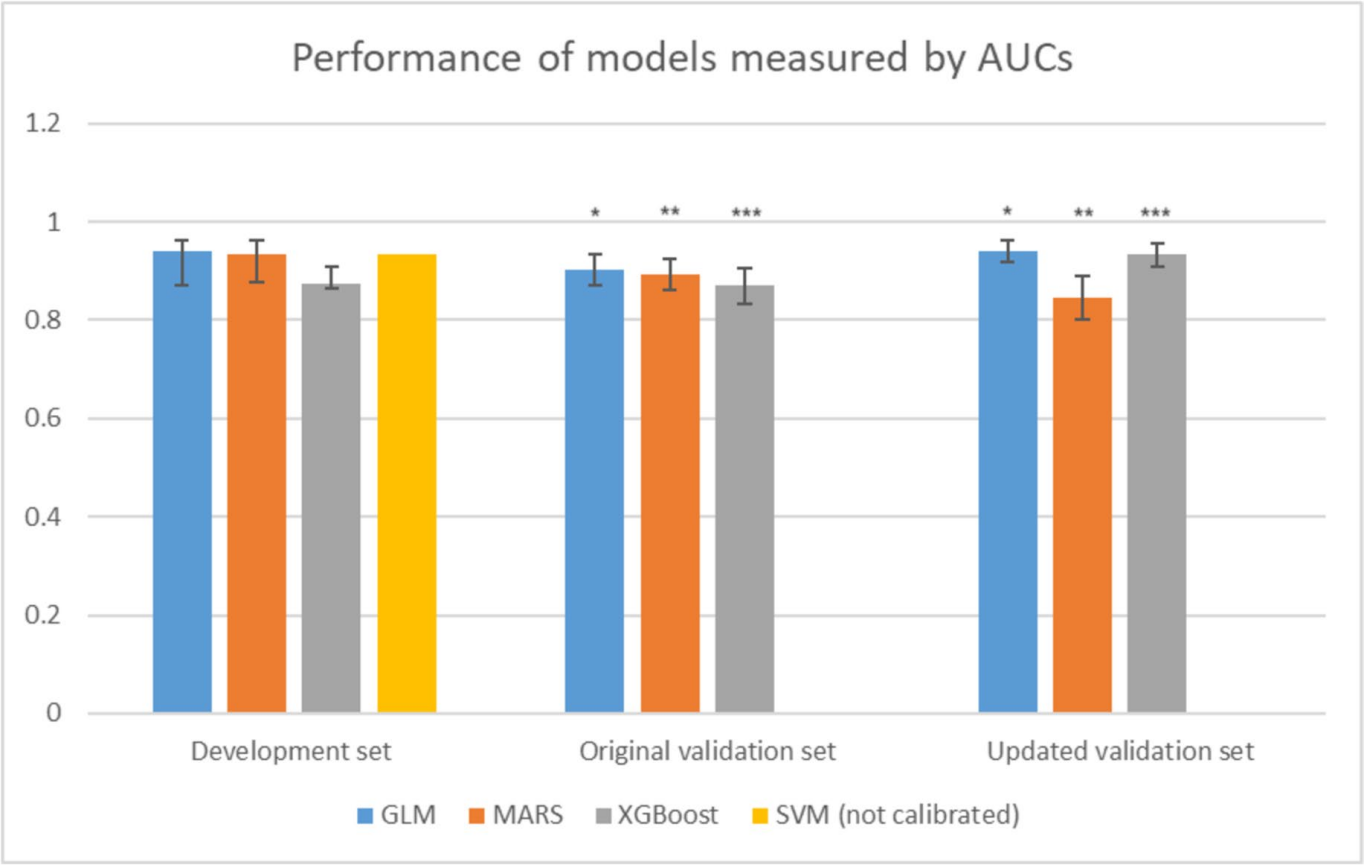
Performance of models measured by AUCs. *AUC* area under the curve, *GLM* logistic regression with elastic net penalty, *MARS* motivation, ability, role perceptions, situational factors, *XGBoost* extreme gradient boosting, *SVM* support vector machine. *stands for statistical significance

In the external validation set, the GLM and XGBoost models showed better performance using the updated software data compared to the original software data: AUC 0.941 (95% CI 0.917–0.961) vs. 0.902 (95% CI 0.872–0.933), *p* < 0.001, and AUC 0.934 (95% CI 0.909–0.956) vs. 0.872 (95% CI 0.833–0.906), *p* < 0.001). The MARS model showed worse performance using the updated software: AUC 0.847 (95% CI 0.802–0.890) vs. 0.894 (95% CI 0.862–0.924), *p* = 0.045) (Fig. 3).

Figure 2 shows the models’ calibration plots and AUC curves for the GLM, MARS, and XGBoost models. Spiegelhalter’s Z statistics showed that GLM (*z* = − 0.410, *p* = 0.341), MARS (*z* = 0.367, *p* = 0.357), and XGBoost (*z* = − 1.948, *p* = 0.026) were well-calibrated models, while SVM (*z* = 2.656, *p* = 0.004) was not calibrated (Figure S1). Of all well-calibrated models, the GLM showed the highest performance (AUC: 0.939, 95% CI 0.872–0.951).

### Insights into model predictions

**Fig. 4:**
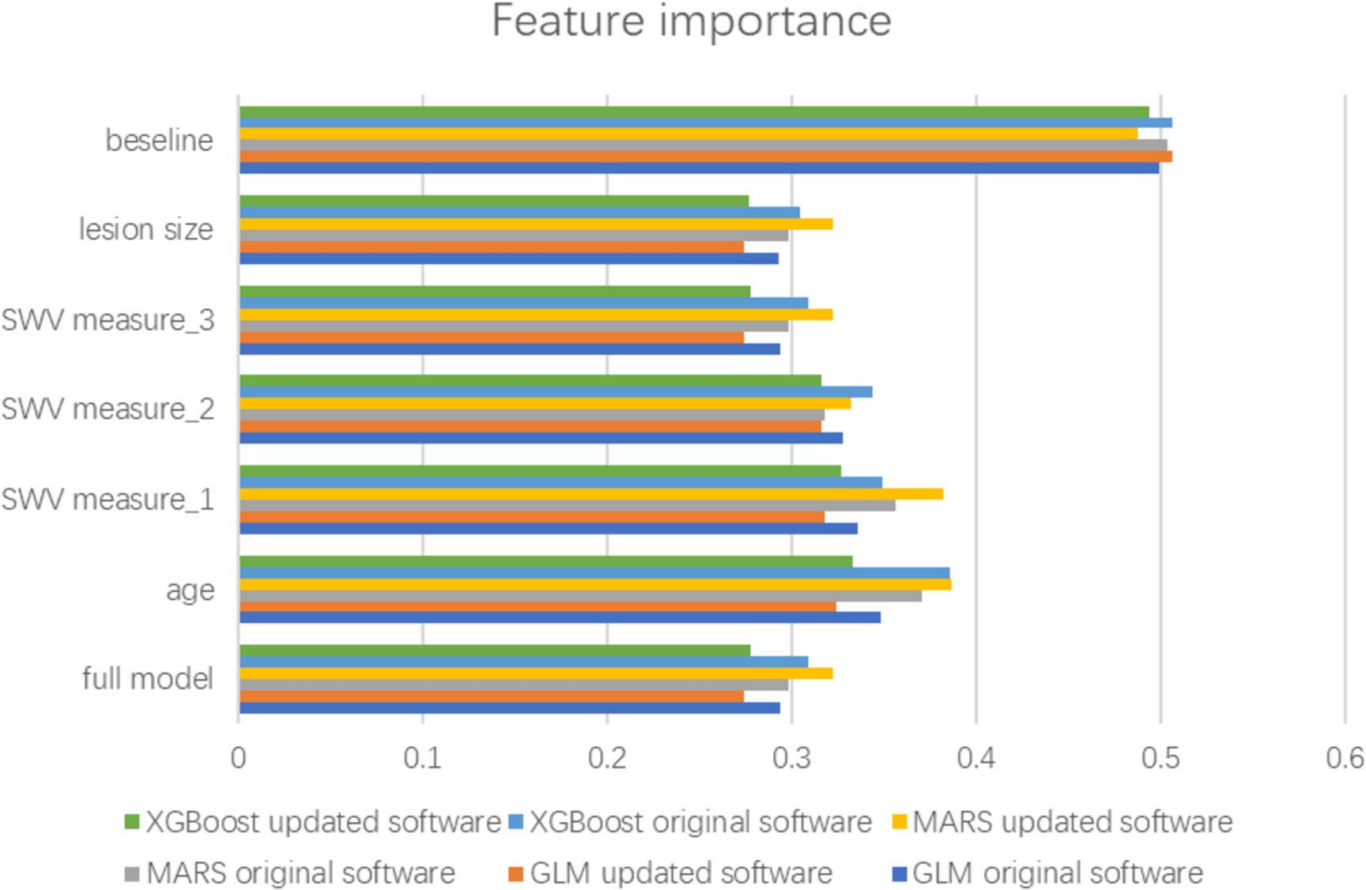
Insights into variable importance of GLM, MARS, and XGBoost models. The baseline represents the loss function when our response values are randomized and indicate the worst-possible loss function value when there is no predictive signal in the data. Feature importance was calculated as 1—mean dropout loss, between 0 and 1, the larger the more important. *AUC* area under the curve, *GLM* logistic regression with elastic net penalty, *MARS* motivation, ability, role perceptions, situational factors, *XGBoost* extreme gradient boosting

Figure 4 illustrates insights into the variable importance for the predictions made by GLM, MARS, and XGBoost models. The top 2 important variables were age and SWV measurement 1 among all models.

Figure S2 shows the decision curve analysis of GLM, MARS, and XGBoost models. Net benefits of these models and the default approaches of treating all (always act) patients or treating none (never act) patients are shown. From 0.19 to 0.61, and 0.67 to 0.89 threshold probabilities, the GLM model validated on the updated software data has a higher net benefit compared to validation on the original software data. From 0.14 to 0.71 threshold probabilities, the XGBoost model validated on the updated software data has a higher net benefit compared to the validation on the original software data. From 0 to 0.33, and 0.35 to 1.0 threshold probabilities, the MARS model validated on the original software data has a higher net benefit compared to the validation on the updated software data.

## Discussion

In this study, we developed machine learning models using multicenter SWE data generated by the original imaging software [10], and externally validated their performance on data generated by both the original updated software versions [9]. All models showed great performance in the development set (AUCs ranging from 0.88 to 0.94), except the SVM model which was not calibrated. In the external validation set, the GLM and XGBoost models showed better performance when validated on the updated software data compared to the original software data. The MARS model showed worse performance when validated on the updated software data. To the best of our knowledge, this is one of the first studies to explore the impact of updated imaging software as a source of data drift and its effect on the generalizability of AI models.

Reproducibility concerns are an emerging topic of high importance in the field of medical AI (image) analyses. Presently, the research community predominantly concentrates on enhancing pre-processing techniques for medical images [20], refining algorithmic structures [21], and incorporating diverse global data spanning different ethnicities and continents, to improve model performance and generalizability [6, 22]. However, once a model is built, we lack monitoring mechanisms to continuously monitor algorithm performance. Data drift may occur over time, leading to impaired generalizability and performance of AI algorithms. Reasons for data drift can be changes in the underlying population (changes in ethnicity, disease stages, or socioeconomic status), changes in the medical standards (e.g. new therapies, diagnostic measures), heterogeneity of hospital information systems [23], and changes in the underlying data itself produced by medical devices (e.g. updated imaging software) [7]. Gulshan et al. developed a deep learning model for the detection of diabetic retinopathy [4], achieving impressive AUC values of 0.99 on two independent American validation datasets in retrospective settings. However, the model's performance declined when prospectively validated across 11 rural clinics in Thailand [24]. One potential explanation for this discrepancy is the difference in the versions of medical imaging software, with more advanced systems being utilized in developed countries compared to those available in rural areas. Hsu et al. developed an ensemble deep learning model for breast mammography screening [25] and validated its performance across the UCLA cohort, the Kaiser Permanente Washington cohort, and the Karolinska Institute cohort. Although the model demonstrated high performance for automated screening mammography interpretation within these specific cohorts, its performance did not generalize well to a more diverse screening population. Whether these varying cohorts utilized the same version of mammography imaging software remains unclear and may have contributed to the observed discrepancies. Prospective validation is to ensure models generalize well on unseen, real-world data, which may have new patterns, variations, and shifts that were not captured in the training data [26]. Concurrent updates in the medical software responsible for data generation have not received adequate attention, so far. Whether model performance remains stable when medical software (and thus the underlying data) is updated, is a knowledge gap that has to be addressed in a rapidly developing field like medical AI.

**Fig. 5:**
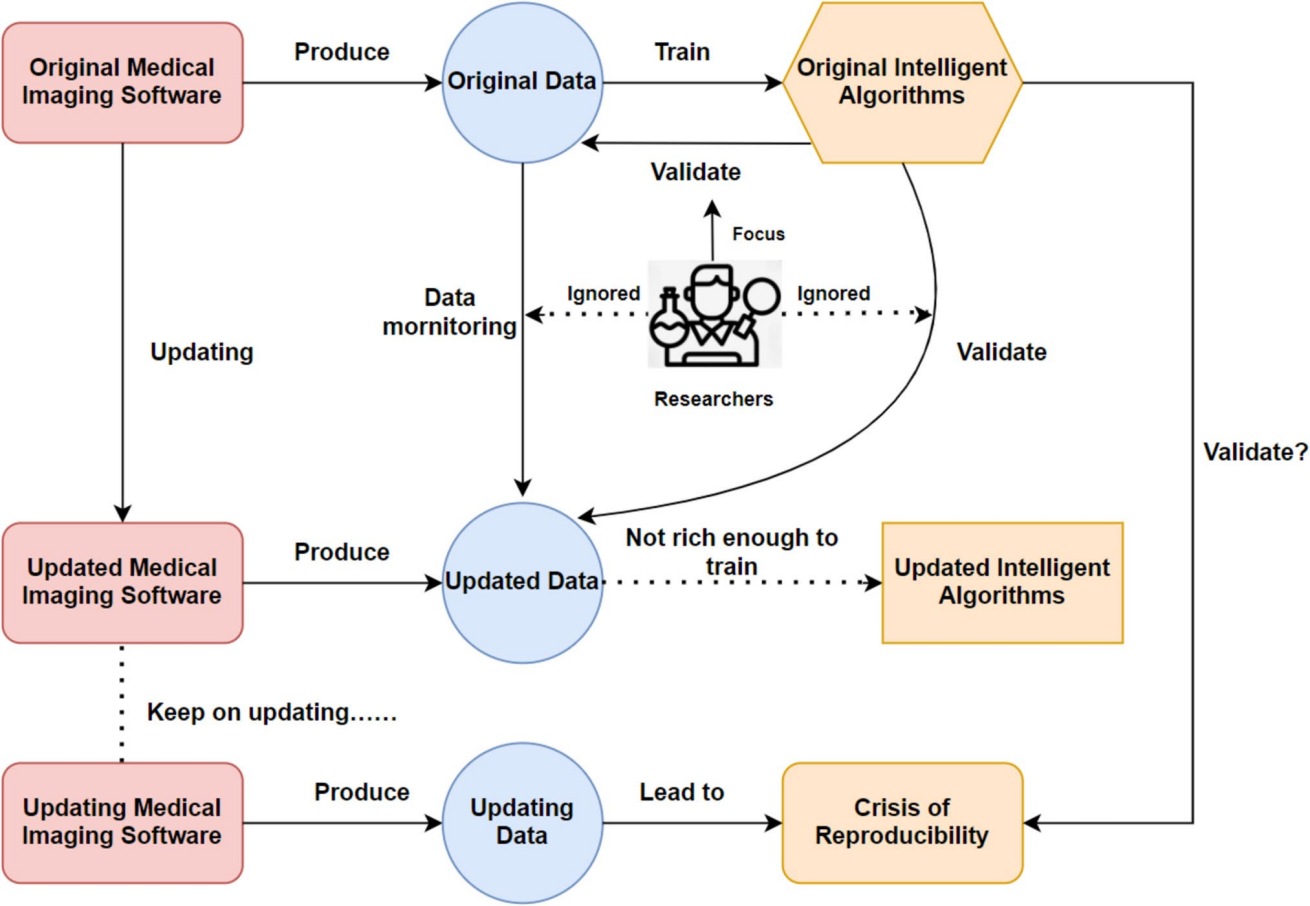
Reproducibility crisis of medical intelligence algorithms

The interplay between AI algorithms, medical imaging software, and data is complex (Fig. 5). AI models are built and trained on data generated by medical imaging software. When updating the imaging software, the data generated by the updated software may differ from the original version. E.g., in this analysis, using the updated software compared to the old version resulted in a significantly higher SWV within the same breast mass. Thus, the performance of AI models may be severely influenced when updating medical software and changing the underlying data. As the U.S. spends about $200 billion on medical software each year, with further increase expected, this is not only relevant from a patient perspective but also from an economic perspective [27]. Besides influencing model performance, updating medical software can have a tremendous impact on users’ workflow as well [28].

In this analysis, we observed statistically significant varying generalizability to the updated software for different models. The GLM model’s performance improved from an AUC value of 0.902 to 0.941 and the XGBoost model’s performance improved from AUC 0.872 to 0.934, whereas the MARS model’s performance dropped from AUC 0.894 to 0.847 and the SVM model was not calibrated upon validation. Possible reasons for the observed difference lay in the model structures themselves: GLM models use a link function to build a linear relationship between the response variables and predictors. They are known for the comparably non-complex structure allowing them to generalize well to new data [29]. XGBoost models repeat training and validating to improve models’ performance. They work well with large, complicated datasets by using various optimizing methods [30]. This may allow them to perform well across versions of software.

This study has limitations. First, this is a retrospective study, potential bias might have affected our findings [31]. Second, elastography measurements were performed on machines from one vendor, allowing conclusions with respect to the generalizability of updated software but not concerning different manufacturer’s machines. Third, the cause of the reproducibility crisis is complex, this study mainly discusses potential data drift because of updated imaging software, while other reasons for data drift, for example, a change in population of interest [6]; are not addressed in this study. This study does also not focus on data monitoring methods to capture data drift [5, 7].

## Conclusion

Updated medical imaging software is a potential source of data drift and its impact on AI algorithms’ performance and generalizability must be considered. In this multicenter study using SWE data, some machine learning models demonstrated great potential to bridge the gap between original software and updated software, whereas others exhibited weak generalizability.

## Supplementary Information

Below is the link to the electronic supplementary material.

Supplementary file1 (DOCX 257 KB)

## Data Availability

Will individual participant data be available (including data dictionaries)? Yes. What data in particular will be shared? Individual participant data that underlie the results reported in this article, after deidentification (text, tables, figures, and appendices). What other documents will be available? None When will data be available (start and end dates)? Immediately following publication. No end date. With whom? Researchers who provide a methodologically sound proposal. For what types of analyses? To achieve the aims in the approved proposal. By what mechanism will data be made available? Proposals should be directed to Michael.golatta@kse-hd.de To gain access, data requestors will need to sign a data access agreement.

## Code availability

We used the open-source “R” programming language (The R Foundation for Statistical Computing). The machine learning frameworks used in this study (TensorFlow and Keras) are available at https://github.com/tensorflow/tensorflow and https://github.com/keras-team/keras.

## References

[1] Health Center for Devices and Radiological (2023) Artificial Intelligence and Machine Learning (AI/ML)-Enabled Medical Devices. FDA

[2] Rajkomar A, Dean J, Kohane I (2019) Machine learning in medicine. N Engl J Med 380:1347–1358. 10.1056/NEJMra1814259 30943338 10.1056/NEJMra1814259

[3] Pfob A, Hillen C, Seitz K et al (2023) Status quo and future directions of digitalization in gynecology and obstetrics in Germany: a survey of the commission Digital Medicine of the German Society for Gynecology and Obstetrics. Arch Gynecol Obstet 309:195–204. 10.1007/s00404-023-07222-2 37755531 10.1007/s00404-023-07222-2 PMC10769997

[4] Gulshan V, Peng L, Coram M et al (2016) Development and validation of a deep learning algorithm for detection of diabetic retinopathy in retinal fundus photographs. JAMA 316:2402. 10.1001/jama.2016.17216 27898976 10.1001/jama.2016.17216

[5] Sedgwick P (2014) Retrospective cohort studies: advantages and disadvantages. BMJ 348:g1072–g1072. 10.1136/bmj.g1072

[6] Pfob A, Sidey-Gibbons C (2022) Systematic bias in medical algorithms: to include or not include discriminatory demographic information? JCO Clin Cancer Inform. 10.1200/CCI.21.00146 35175859 10.1200/CCI.21.00146

[7] George R, Ellis B, West A et al (2023) Ensuring fair, safe, and interpretable artificial intelligence-based prediction tools in a real-world oncological setting. Commun Med 3:88. 10.1038/s43856-023-00317-6 37349541 10.1038/s43856-023-00317-6 PMC10287624

[8] Berg WA, Cosgrove DO, Doré CJ et al (2012) Shear-wave elastography improves the specificity of breast US: the BE1 multinational study of 939 masses. Radiology 262:435–449. 10.1148/radiol.11110640 22282182 10.1148/radiol.11110640

[9] Barr RG, Engel A, Kim S et al (2023) Improved breast 2D SWE algorithm to eliminate false-negative cases. Invest Radiol 58:703–709. 10.1097/RLI.0000000000000972 36939607 10.1097/RLI.0000000000000972

[10] Pfob A, Sidey-Gibbons C, Barr RG, et al (2022) Intelligent multi-modal shear wave elastography to reduce unnecessary biopsies in breast cancer diagnosis (INSPiRED 002): a retrospective, international, multicentre analysis. In: European Journal of Cancer. Elsevier Ltd, pp 1–14 10.1016/j.ejca.2022.09.018 36283244

[11] Liu Y, Chen P-HC, Krause J, Peng L (2019) How to read articles that use machine learning. JAMA 322:1806. 10.1001/jama.2019.16489 31714992 10.1001/jama.2019.16489

[12] Pfob A, Lu S-C, Sidey-Gibbons C (2022) Machine learning in medicine: a practical introduction to techniques for data pre-processing, hyperparameter tuning, and model comparison. BMC Med Res Methodol 22:282. 10.1186/s12874-022-01758-8 36319956 10.1186/s12874-022-01758-8 PMC9624048

[13] Cohen JF, Korevaar DA, Altman DG et al (2016) STARD 2015 guidelines for reporting diagnostic accuracy studies: explanation and elaboration. BMJ Open. 10.1136/bmjopen-2016-012799 28137831 10.1136/bmjopen-2016-012799 PMC5128957

[14] Collins GS, Reitsma JB, Altman DG, Moons K (2015) Transparent reporting of a multivariable prediction model for individual prognosis or diagnosis (TRIPOD): the TRIPOD Statement. BMC Med 13:1. 10.1186/s12916-014-0241-z 25563062 10.1186/s12916-014-0241-z PMC4284921

[15] Vickers AJ, Woo S (2022) Decision curve analysis in the evaluation of radiology research. Eur Radiol 32:5787–5789. 10.1007/s00330-022-08685-8 35348862 10.1007/s00330-022-08685-8

[16] Venkatraman ES (2000) A permutation test to compare receiver operating characteristic curves. Biometrics 56:1134–1138. 10.1111/j.0006-341x.2000.01134.x 11129471 10.1111/j.0006-341x.2000.01134.x

[17] Newcombe RG (1998) Two-sided confidence intervals for the single proportion: comparison of seven methods. Stat Med 17:857–872. 10.1002/(sici)1097-0258(19980430)17:8%3c857::aid-sim777%3e3.0.co;2-e 9595616 10.1002/(sici)1097-0258(19980430)17:8<857::aid-sim777>3.0.co;2-e

[18] Spiegelhalter DJ (1986) Probabilistic prediction in patient management and clinical trials. Stat Med 5:421–433. 10.1002/sim.4780050506 3786996 10.1002/sim.4780050506

[19] Harrell FE, Lee KL, Mark DB (1996) Multivariable prognostic models: issues in developing models, evaluating assumptions and adequacy, and measuring and reducing errors. Stat Med 15:361–387. 10.1002/(SICI)1097-0258(19960229)15:4%3c361::AID-SIM168%3e3.0.CO;2-4 8668867 10.1002/(SICI)1097-0258(19960229)15:4<361::AID-SIM168>3.0.CO;2-4

[20] Isaksson LJ, Summers P, Mastroleo F et al (2023) Automatic segmentation with deep learning in radiotherapy. Cancers (Basel) 15:4389. 10.3390/cancers15174389 37686665 10.3390/cancers15174389 PMC10486603

[21] Papanastasiou G, Dikaios N, Huang J et al (2023) Is attention all you need in medical image analysis? IEEE J Biomed Health Inform, A review. 10.1109/JBHI.2023.3348436 10.1109/JBHI.2023.3348436 38157463

[22] Obermeyer Z, Powers B, Vogeli C, Mullainathan S (2019) Dissecting racial bias in an algorithm used to manage the health of populations. Science (1979) 366:447–453. 10.1126/science.aax2342 10.1126/science.aax2342 31649194

[23] Pfob A, Griewing S, Seitz K et al (2023) Current landscape of hospital information systems in gynecology and obstetrics in Germany: a survey of the commission Digital Medicine of the German Society for Gynecology and Obstetrics. Arch Gynecol Obstet 308:1823–1830. 10.1007/s00404-023-07223-1 37740792 10.1007/s00404-023-07223-1 PMC10579143

[24] Beede E, Baylor E, Hersch F, et al (2020) A Human-Centered Evaluation of a Deep Learning System Deployed in Clinics for the Detection of Diabetic Retinopathy. In: Proceedings of the 2020 CHI Conference on Human Factors in Computing Systems. ACM, New York, NY, USA, pp 1–12

[25] Hsu W, Hippe DS, Nakhaei N et al (2022) External validation of an ensemble model for automated mammography interpretation by artificial intelligence. JAMA Netw Open. 10.1001/jamanetworkopen.2022.42343 36409497 10.1001/jamanetworkopen.2022.42343 PMC9679879

[26] Cai L, Pfob A (2024) Artificial intelligence in abdominal and pelvic ultrasound imaging: current applications. Abdominal Radiology 10.1007/s00261-024-04640-x PMC11947003 39487919

[27] Lindgren L, Kesselheim AS, Kramer DB (2022) The right to repair software-dependent medical devices. J Law Med Ethics 50:857–859. 10.1017/jme.2023.28 36883383 10.1017/jme.2023.28

[28] Ahlbrandt J, Bott C, Moll P et al (2015) Version changes in medical software: proposing minimal requirements for release notes and a version number convention: an operators’ point of view. Stud Health Technol Inform 210:210–214 25991132

[29] Faraway JJ (2010) Generalized Linear Models. In: Peterson P, Baker E, McGaw B (eds) International Encyclopedia of Education, 3rd edn. Elsevier, Oxford, pp 178–183

[30] Ethem A (2020) Introduction to Machine Learning. MIT Press

[31] Talari K, Goyal M (2020) Retrospective studies: utility and caveats. J Royal Coll Phys Edinb 50:398–402. 10.4997/jrcpe.2020.409 10.4997/JRCPE.2020.409 33469615

