# Supplementary material for: The impact of updated imaging software on the performance of machine learning models for breast cancer diagnosis: a multi-center, retrospective study
Source: Arch Gynecol Obstet. 2025 Jan 30;312(1):139–47. doi: 10.1007/s00404-024-07901-8 (PMC12176987; doi:10.1007/s00404-024-07901-8)
Supplement: Supplementary file 1 — Supplementary file1 (DOCX 257 KB) [file 404_2024_7901_MOESM1_ESM.docx]

**Supplement for article**

**The impact of updated imaging software on the performance of machine learning models for breast cancer diagnosis. A multi-center, retrospective study**

We provide

1. a checklist informed by recent guidelines on machine learning in medicine
2. different model’s AUC value with 95% confidence interval and corresponding hyperparameters
3. calibration plot of support vector machine (SVM)
4. Decision curve analysis of GLM, MARS, and XGBoost models.

Table 1. A checklist informed by recent guidelines on machine learning in medicine according to Liu et al^1^

| Aspect of study |  | Our study | Considerations |
| --- | --- | --- | --- |
| Summary of prediction task | | Identify malignant breast lesions | What is the clinical utility of having these predictions available? |
| Data | Input | 5: age, lesion size, three times of lesion’s shear wave velocity measurements | Are these data easily obtainable in routine clinical workflows? |
|  | Output | 2-class: malignant *vs.* benign | Is the prediction clinically relevant? Is this prediction at the right granularity, such as a meaningful number of patient risk categories? |
|  | Label | Pathological evaluation of the breast biopsy specimen served as the gold standard for the definition of breast lesions. All malignant lesions were biopsy proven with final pathology, tumor grade, and tumor markers recorded. | Is this an accepted grading scale and is the reference standard reliable? |
|  | Patient population | For development set: Women aged 18 years or older who presented as with a suspicious or indeterminate single breast mass of diameters between 0.5 and 5cm in B-mode ultrasound from February 2016 to March 2019 were included in this study.  For external validation set: Included patients scheduled for a screening or diagnostic breast ultrasound from April 25, 2019 to May 2, 2022 performed on an ultrasound system. | What are the patient population and inclusion/exclusion criteria? What are the numbers of patients with  each label and other characteristics? |
|  | Development / Validation split | We used international, multicenter SWE data (NCT 02638935)^2^as the development set, of which included 1288 cases. We used single-center SWE data as the external validation set, which was obtained by original software and new FDA approved updated software,^3^ we included 385 cases in this study. | Do any patients appear in both development and validation sets? Is this a strong study design with respect to evaluation of generalization? |
|  | Amount of data in the development set | 1288 cases (368 events) in the training and internal testing data | Is this sufficient to develop the ML model, given its complexity? |
|  | Amount of data in the validation set | 385 cases (124 events) in the validation set | Is this sufficient to have confidence in the generalizability of the results and any clinically important subgroups? |
| Machine Learning (ML) | Method | Logistic regression with elastic net penalty (GLM), motivation, ability, role perceptions and situational factors (MARS), support vector machine (SVM), extreme gradient boosting (XGBoost) | Is this a standard or customized method? What are the parameters and how many are there? |
|  | Training process | Random initialization, single 2-class prediction | Was transfer learning (eg, preinitialization), or multi-task learning (eg, multiple predictions) used to help training? |
|  | Data augmentation | Not applicable | Data augmentation typically helps performance and generalization. Was this done and appropriate for this data type? |
|  | Hyperparameters that were optimized | GLM: Alpha determines the strength of the regularization. Lambda controls how strongly coefficients are pushed toward zero.  SVM:  Gamma:a small gamma means a Gaussian with a large variance; large gamma leads to high bias and low variance models, and vice-versa.  C: p**arameter of the soft margin cost function, which controls the influence of each individual support vector**  **MARS**  **degree: the maximum degree of interactions (Rarely is there any benefit in assessing greater than 3-rd degree interactions)**  **nprune: the number of terms retained in the final model**  XGBoost  max_depth:Controls the maximum depth of the trees.  eta: range [0,1] Learning (or shrinkage) parameter.  gamma: range [0,endless] Minimum loss reduction required to make a further partition on a leaf node of the tree. The larger gamma is, the more conservative the algorithm will be.  colsample_bytree: range [0,1] colsample_bytree is the subsample ratio of columns when constructing each tree. Subsampling occurs once for every tree constructed.  min child weight: Minimum sum of instance weight (hessian) needed in a child.  subsample: range [0,1] Subsample ratio of the training instances. | What were the hyperparameter s? |
|  | Use of tuning set | Yes, 10-fold cross validation | Was a separate tuning set (independent of the final validation set) used for hyperparameter tuning? |
|  | Time taken to apply model per data point | Not reported | Is this amount of time feasible in the context of routine clinical workflows? |
| Evaluation | Dataset inclusion/exclusion criteria | Please find in the original article Methods$Study design | Were any data excluded based on ML predictions? If so, why and does this skew the results? |
|  | Performance metric | Performance of models was mainly evaluated using the area under the receiving operator curve (AUROC), Confidence intervals for AUROC were computed with 2000 stratified bootstrap replicates).  Decision curve analysis (DCA) to better illustrate the benefits of clinical application of the models.^4^  Calibration: calibration plots (observed vs. predicted probability) and Spiegelhalter’s Z statistics.^5^ | Is this standard? What is “random” performance (eg, 0 or 0.5), and what is perfect (eg, 1)? Is this appropriate given the incidence or prevalence of the predicted label? How were the operating points selected? |
|  | Was this an independent validation set? | Yes, external independent validation set | Was this final validation set used to make any ML model development decisions? Including but not limited to hyperparameter tuning, neural network “checkpoint” selection, method selection, etc |
|  | Human comparison metric | Not applicable | Is this a fair comparison: eg, does the human grader have sufficient training (eg, years post residency), information (eg, other clinical variables) and time (eg, comparable with routine practice)?1 Are the statistics (confidence intervals etc.) present and appropriate? |
|  | Human comparator | Not applicable | Were there any deviations from standard practice? eg, were the human graders provided sufficient time and with the full-resolution image? |
|  | Human vs ML model performance | Not applicable | Based on prior literature, are the human performance sensible? Do they over- or under-represent human performance? |
|  | Performance gap in development-validation | Models were built using data generated by original software. Performance was validated by using data generated by original software and updated software. | What was the gap between tuning and validation, and does this suggest good generalizability? |
|  | Subgroup / sensitivity analysis | No potential confounding factors to report | Are there any potential confounding factors that should be examined more closely (eg, image capturing device manufacturer or model, etc.)? |

Table 2. different model’s AUC value with 95% confidence interval in the development set and corresponding hyperparameters

| Models | AUC | 95%CI | Hyperparameters |
| --- | --- | --- | --- |
| GLM | 0.939 | 0.872-0.961 | alpha = 0.180, lambda = 0.031 |
| SVM | 0.935 | 0.864-0.914 | degree = 1, scale = 0.035, C = 0.082 |
| MARS | 0.935 | 0.877-0.908 | degree = 1, nprune = 6 |
| XGBoost | 0.875 | 0.866-0.909 | eta = 0.2, max depth = 5, gamma = 5, colsample by tree = 0.8, min child weight = 5, subsample =1, nrounds = 5 |

Abbreviations: AUC, area under the curve; CI, confidence interval; GLM, logistic regression with elastic net penalty; MARS, motivation, ability, role perceptions, situational factors; XGBoost, extreme gradient boosting.

Figure 1. Calibration plot of SVM model


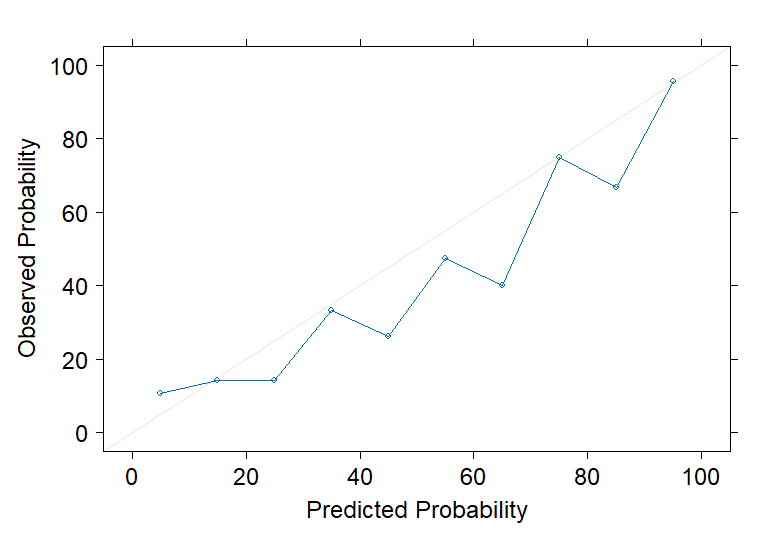


Figure 2 Decision curve analysis of GLM, MARS, and XGBoost models.


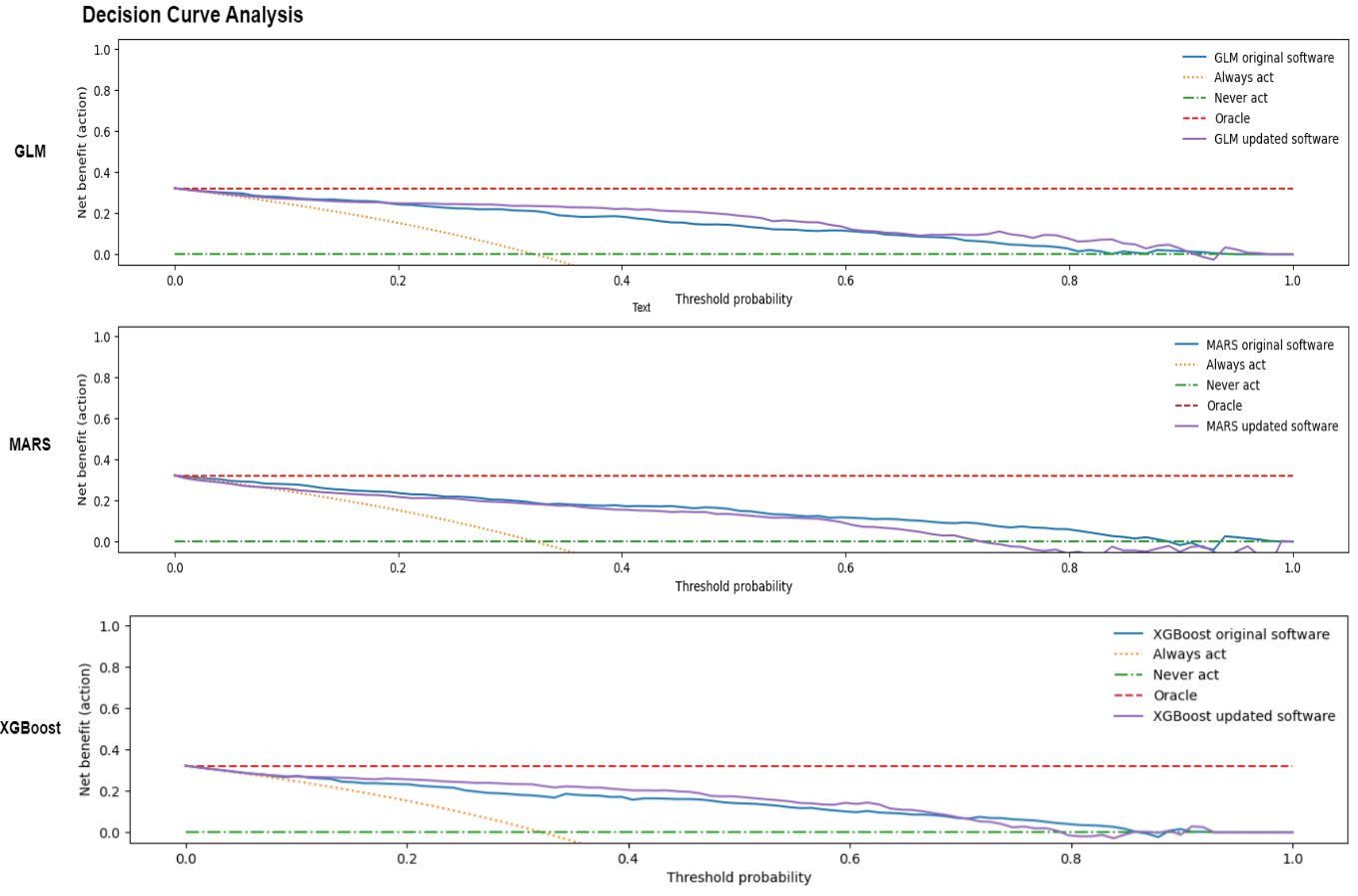


Abbreviations: AUC, area under the curve; GLM, logistic regression with elastic net penalty; MARS, motivation, ability, role perceptions, situational factors; XGBoost, extreme gradient boosting.

**References**

1 Liu Y, Chen P-HC, Krause J, Peng L. How to Read Articles That Use Machine Learning. *JAMA* 2019; **322**: 1806.

2 Pfob A, Sidey-Gibbons C, Barr RG, *et al.* Intelligent multi-modal shear wave elastography to reduce unnecessary biopsies in breast cancer diagnosis (INSPiRED 002): a retrospective, international, multicentre analysis. In: European Journal of Cancer. Elsevier Ltd, 2022: 1–14.

3 Barr RG, Engel A, Kim S, Tran P, De Silvestri A. Improved Breast 2D SWE Algorithm to Eliminate False-Negative Cases. *Invest Radiol* 2023; **58**: 703–9.

4 Vickers AJ, Woo S. Decision curve analysis in the evaluation of radiology research. *Eur Radiol* 2022; **32**: 5787–9.

5 Spiegelhalter DJ. Probabilistic prediction in patient management and clinical trials. *Stat Med* 1986; **5**: 421–33.
